# Bat-associated ticks as a potential link for vector-borne pathogen transmission between bats and other animals

**DOI:** 10.1371/journal.pntd.0012584

**Published:** 2024-10-25

**Authors:** Tamara Szentiványi, Nóra Takács, Attila D. Sándor, Áron Péter, Sándor A. Boldogh, Dávid Kováts, Jeffrey T. Foster, Péter Estók, Sándor Hornok

**Affiliations:** 1 Pathogen and Microbiome Institute, Northern Arizona University, Flagstaff, Arizona, United States of America; 2 Department of Parasitology and Zoology, University of Veterinary Medicine, Budapest, Hungary; 3 HUN-REN-UVMB Climate Change: New Blood-Sucking Parasites and Vector-Borne Pathogens Research Group, Budapest, Hungary; 4 STAR-UBB Institute, Babes-Bolyai University, Cluj-Napoca, Romania; 5 Aggtelek National Park Directorate, Jósvafő, Hungary; 6 Hungarian Biodiversity Research Society, Budapest, Hungary; 7 Eszterházy Károly Catholic University, Eger, Hungary; Egerton University - Njoro Campus: Egerton University, KENYA

## Abstract

**Background:**

Potentially zoonotic pathogens have been previously detected in bat-associated ticks, but their role in disease transmission and their frequency of feeding on non-bat hosts is poorly known.

**Methodology/Principal findings:**

We used molecular blood meal analysis to reveal feeding patterns of the bat-associated tick species *Ixodes ariadnae*, *I*. *simplex*, and *I*. *vespertilionis* collected from cave and mine walls in Central and Southeastern Europe. Vertebrate DNA, predominantly from bats, was detected in 43.5% of the samples (70 of 161 ticks) but in these ticks we also detected the DNA of non-chiropteran hosts, such as dog, *Canis lupus familiaris*, wild boar, *Sus scrofa*, and horse, *Equus caballus*, suggesting that bat-associated ticks may exhibit a much broader host range than previously thought, including domestic and wild mammals. Furthermore, we detected the zoonotic bacteria *Neoehrlichia mikurensis* in bat ticks for the first time, and other bacteria, such as *Bartonella* and *Wolbachia*.

**Conclusions/Significance:**

In the light of these findings, the role of bat ticks as disease vectors should be urgently re-evaluated in more diverse host systems, as they may contribute to pathogen transmission between bats and non-chiropteran hosts.

## Author summary

Ticks found on bats might carry diseases that can be important for both animal and human health. In this study we collected three species of bat ticks and used molecular methods to demonstrate their feeding habits and to explore the pathogens they carry. We found that most of the ticks had been feeding on bats, but we also found evidence of blood meals from domestic and wild animals, such as dogs, wild boars, and horses. We also found several bacteria in these ticks that can infect or potentially infect humans and animals. These results indicate that bat ticks could be a potential link between bats and non-bat hosts and may help spread diseases between them. Learning about how ticks interact with different host species is critical to understanding the ecology and spread of wildlife disease.

## Introduction

Ticks (Acari: Ixodida) are the main vectors of a variety of historical and emerging infectious pathogens to humans, domestic animals, and wildlife species, with nearly 1000 tick species currently known worldwide [1,2]. Tick bites on humans and tick-borne diseases have increased over the past decade and have been responsible for most emerging vector-borne diseases [3,4]. Tick populations are increasing in many regions and are expanding their geographical distributions [5]. Habitat loss, landscape modification, climate change, and increased interactions among wildlife, domestic animals, and humans may contribute to the spread of several new and emerging tick-borne pathogens [6]. Ticks are currently recognized as the most important vectors transmitting zoonotic bacterial pathogens to humans [7].

Most terrestrial vertebrate species, including bats, harbor ectoparasitic arthropods, as exemplified by ticks. Bats are known hosts of several tick-borne bacteria with zoonotic potential [8]. Bat-associated ticks have been found to harbor a wide variety of pathogens, some with high veterinary and public health importance, including species from the following genera: *Anaplasma*, *Bartonella*, *Borrelia*, *Mycoplasma*, *Rickettsia* and *Babesia* [9–15]. Despite their importance, ubiquity, and human health relevance, potential transmission routes of these pathogens from bats remain poorly known. For instance, human pathogenic *Borrelia* spp. have only been recently detected for the first time in bat ticks [12]. On the other hand, bat-associated *Borrelia* spp. can occasionally infect humans as well [16]. Additionally, bat-associated *Bartonella* spp. and antibodies have been also observed in humans in Africa and in Europe, suggesting their potential to induce disease [17,18].

Host specificity can greatly vary among ticks, which can determine their role in disease transmission [19]. Similarly to other tick species, bat-associated ticks show different levels of host specificity depending on their species [20]. Among hard ticks (Ixodida: Ixodidae) of bats, *Ixodes ariadnae* is most often found on *Myotis* species [21], whereas *I*. *simplex* is considered a specific parasite of *Miniopterus schreibersii* [22], and *I*. *vespertilionis* predominates on *Rhinolophus* spp. [20,23]. However, limited information is available on the alternate hosts of bat-associated ticks besides bats. The latter two ixodid species, as well as soft ticks (Ixodida: Argasidae) of bats, such as *Chiropterargas boueti*, *Carios kelleyi*, *C*. *vespertilionis*, *Secretargas transgariepinus*, and *Reticulinasus salahi* occasionally feed on humans [24–26], which has been observed in Europe, North and South America, with an increasing number of accounts over past two decades [24,25,27–30]. These data suggest that bat-associated tick species may opportunistically feed on non-chiropteran hosts. However, the frequency of such behavior is currently unknown, most importantly because the primary source of host preference data comes from field observation of ticks infesting bats [20,24]. While these data are highly valuable, they lack information of the complete host spectrum and occasional feeding on other hosts.

Molecular blood meal analysis is a useful tool to identify host(s) used by previous life cycle stage(s) of off-host collected ticks in the wild. For instance, the soft tick species, *C*. *kelleyi*, a specific parasite of bats in North America, is occasionally found in human settlements, usually when bats occupy attics or other parts of the home, and have been found to feed on humans and dogs based on blood meal analysis [25,31]. Two generalist and widespread Palearctic hard tick species, *I*. *ricinus* and *I*. *persulcatus*, that frequently feed on humans also feed on bats based on molecular blood meal analysis, suggesting the public health importance of such events [32,33]. Although these data are limited, they suggest that bat-associated ticks may play a hitherto neglected link between bats and non-chiropteran hosts, implying their potential role in the zoonotic transmission of bat-derived vector-borne pathogens.

In this work, we aimed to explore host spectrum and pathogen diversity of bat-associated ticks, to better understand bat tick feeding biology and the pathogen pathways and potential disease emergence threats across host species.

## Materials and methods

### Ethics statement

This work was conducted under animal handling permits TMF/513/1/2004.

### Tick collection

Ticks were collected in caves and mines between 2016 and 2023, at nine locations in Hungary and 12 locations in Romania (S1 Table and S1 Fig). All ticks were collected near bat colonies from cave and mine walls. Importantly, we did not use ticks that were directly collected from bats in this work, therefore this work did not involve handling of live bats. Using forceps or collection by hand, ticks were placed to 70% ethanol until further processing. Tick identification was carried out based on morphological characteristics by authors SH and TS using standard identification keys [21–23,34].

### Data and molecular analysis of blood meal and pathogen presence

A total of 161 ticks belonging to three species were used to test blood meal presence and pathogen occurrence. Before DNA extraction, each tick was given a surface sterilization with 10% sodium-hypochlorite solution. Ticks were individually and mechanically homogenized using sterilized scissors. DNA extraction was done from individual ticks using the manufacturer’s protocol of tissue extraction (QIAamp DNA Mini Kit, Qiagen, Germany). To detect host DNA we targeted a vertebrate specific Cytochrome c Oxidase I (COI) region for blood meal analysis with conventional PCR, using the primers: SFF_145f (5’—GTHACHGCYCAYGCHTTYGTAATAAT—3’) and SFF_351r (5’ -CTCCWGCRTGDGCWAGRTTTCC- 3’), following the previously described protocol [35]. Pathogen detection was also performed using conventional PCR, screening for Anaplasmataceae, *Bartonella* spp., *Borrelia burgdorferi* s.l., *Rickettsia* spp., and piroplasms. Primers and PCR conditions are in the S1 Appendix. We repeated PCR and sequencing whenever we obtained low quality products or sequences. PCR products were visualized on 1.5% agarose gel and were sent for sequencing to Eurofins BIOMI Kft., Gödöllő, Hungary. New sequences were submitted to GenBank (Accession numbers: PQ432800—PQ432803, PQ444043—PQ444051, PQ444181—PQ444185).

Multiple sequence alignments were done using ClustalW software [36]. Evolutionary analyses and tree visualization were conducted in MEGA 11 [37]. We performed the Maximum Likelihood method based on the Kimura 2-parameter model [38], and the Hasegawa-Kishino-Yano model with gamma distribution and invariant sites [39], based on 1000 bootstrap replicates. We applied NCBI BLAST for sequence identification and reference sequences were obtained from GenBank (S2 Table). RStudio was used to visualize data, using ggplot2 package [40,41]. Spatial data for S1 Fig were obtained from the Natural Earth dataset (available at: https://www.naturalearthdata.com) using QGIS 3.24 [42,43].

## Results

### Blood meal analysis and host range

**Fig 1:**
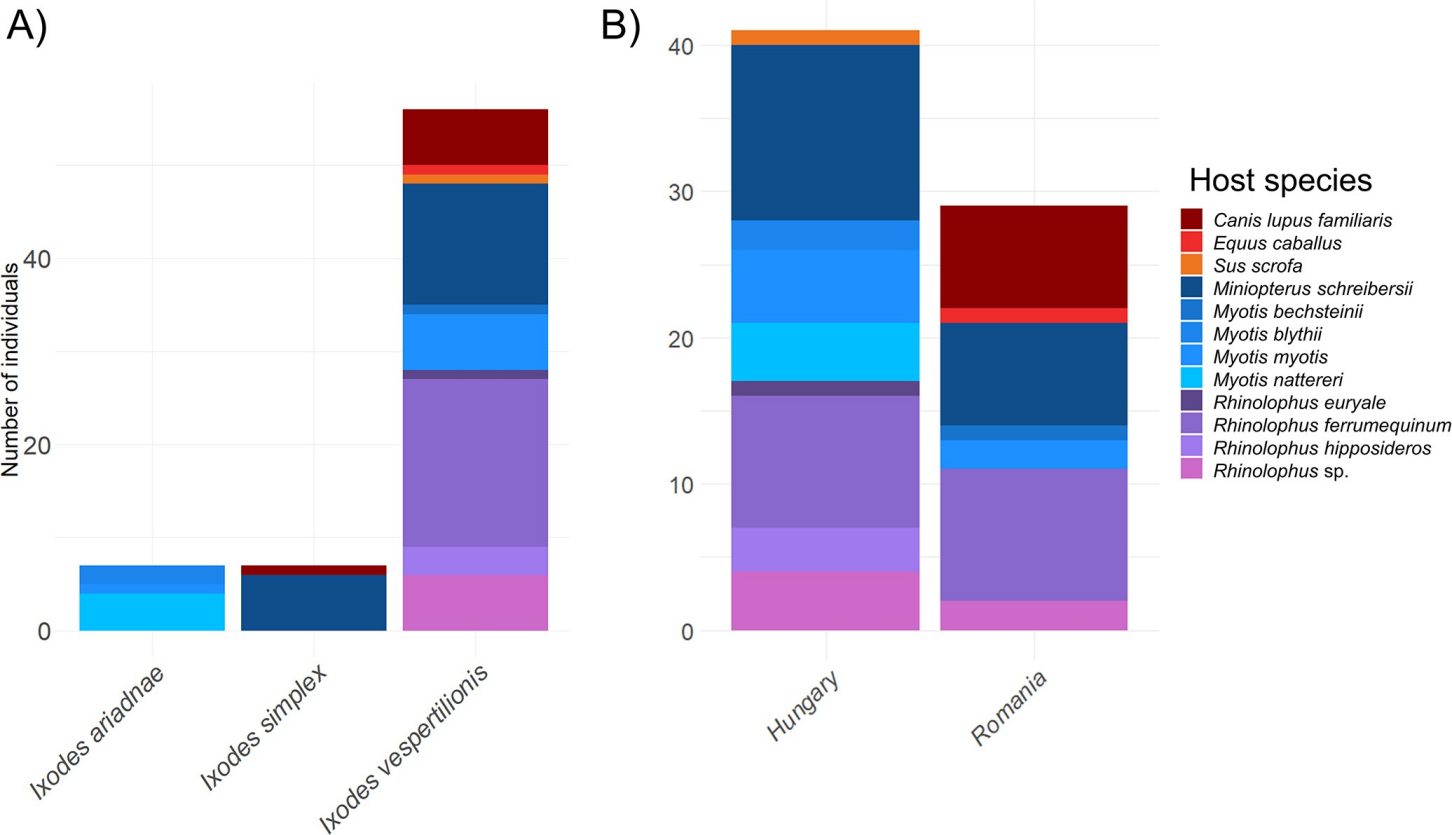
Host blood meal presence in positive tick samples (n = 70) according to tick species (A) and country of origin (B). Negative samples and samples with insufficient sequence quality were excluded.

We tested three bat-associated tick species, *I*. *ariadnae* (n = 11), *I*. *simplex* (n = 9), and *I*. *vespertilionis* (n = 141). Other tick species were not found at the sampling locations. We screened one larva, nine nymphs, 87 adult female ticks, and 64 adult male ticks (S1 Table). We detected DNA in blood meals from vertebrate hosts in 43.5% (n = 70) of the 161 samples, including in *I*. *ariadnae* (n = 7/11, 63.6%), *I*. *simplex* (n = 7/9, 77.8%), and *I*. *vespertilionis* (n = 56/141, 39.7%). Samples were considered negative when they had an apparent positive result during PCR (i.e., band of appropriate size), but no sequence data could be generated (n = 27/161, 16.8%). A total of 64 (39.8%) samples had PCR negative result for vertebrate blood meal. *Ixodes ariadnae* contained the DNA of *M*. *blythii* (n = 2), *M*. *myotis* (n = 1) and *M*. *nattereri* (n = 4); while *I*. *simplex* showed the presence of host DNA from *M*. *schreibersii* (n = 6) and a non-bat host, *Canis lupus familiaris* (n = 1, Fig 1). Furthermore, *I*. *vespertilionis* indicated the broadest spectrum of host feeding, including *M*. *schreibersii* (n = 13), *M*. *bechsteinii* (n = 1), *M*. *myotis* (n = 6), *Rhinolophus euryale* (n = 1), *R*. *ferrumequinum* (n = 18), *R*. *hipposideros* (n = 3), *Rhinolophus* sp. (n = 6), and non-bat hosts of *C*. *lupus familiaris* (n = 6), *Equus caballus* (n = 1), and *Sus scrofa* (n = 1) (Fig 1). One larval stage (EW 82) of *I*. *vespertilionis* was collected from the same location in May and showed the presence of *R*. *ferrumequinum* DNA. All nine nymphs included in this study were identified as *I*. *vespertilionis* tick species and were all collected in Hungary. Except for a single individual tick (EW83) that had fed on *R*. *ferrumequinum*, all of the other eight nymphs contained *M*. *schreibersii* DNA (S1 Table). Host DNA was also detected in both sexes, including females of *I*. *ariadnae* (n = 3/5, 60%), *I*. *simplex* (n = 4/4, 100%), and *I*. *vespertilionis* (n = 25/78, 32%); and males of *I*. *ariadnae* (n = 4/6, 66.7%), *I*. *simplex* (n = 3/5, 60%), and *I*. *vespertilionis* (n = 21/53, 39.6%). Overall, ticks collected both in Hungary (41/93, 44%) and Romania (29/68, 42.6%) had detectable amounts of host DNA (Fig 1).

### Pathogen presence

**Fig 2:**
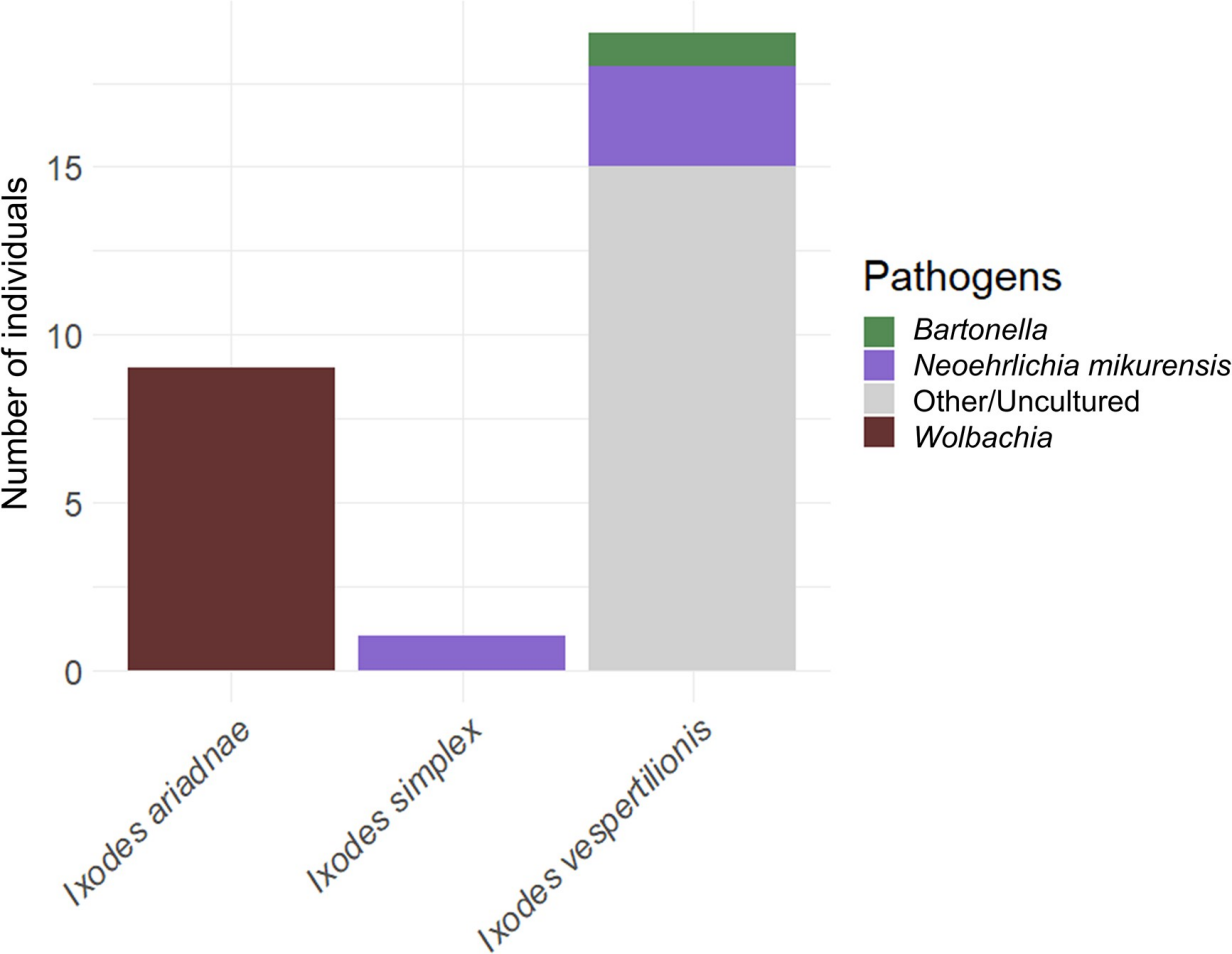
Presence of pathogens across tick species. Samples that could not be identified due to low similarity are indicated as Other. Uncultured bacterial sequences (labeled as Uncultured) showed high similarity to samples in GenBank, however their identity could not be determined.

**Fig 3:**
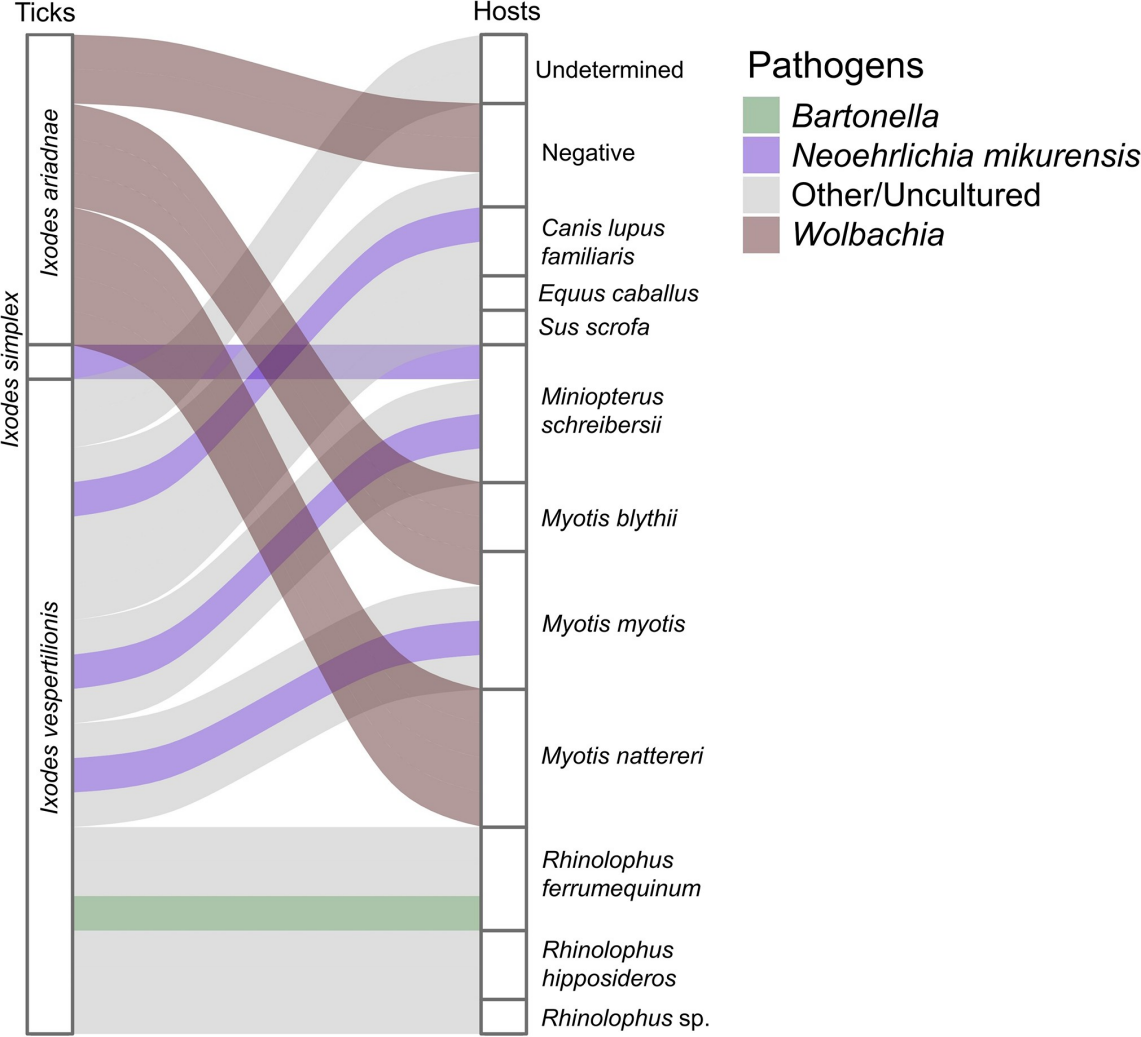
Association between bat ticks (left columns) and identified hosts (right columns) based on molecular blood meal analysis. Associated pathogens are indicated with different colors. The height of the bars represents the relative abundance of the groups within each network level.

**Fig 4:**
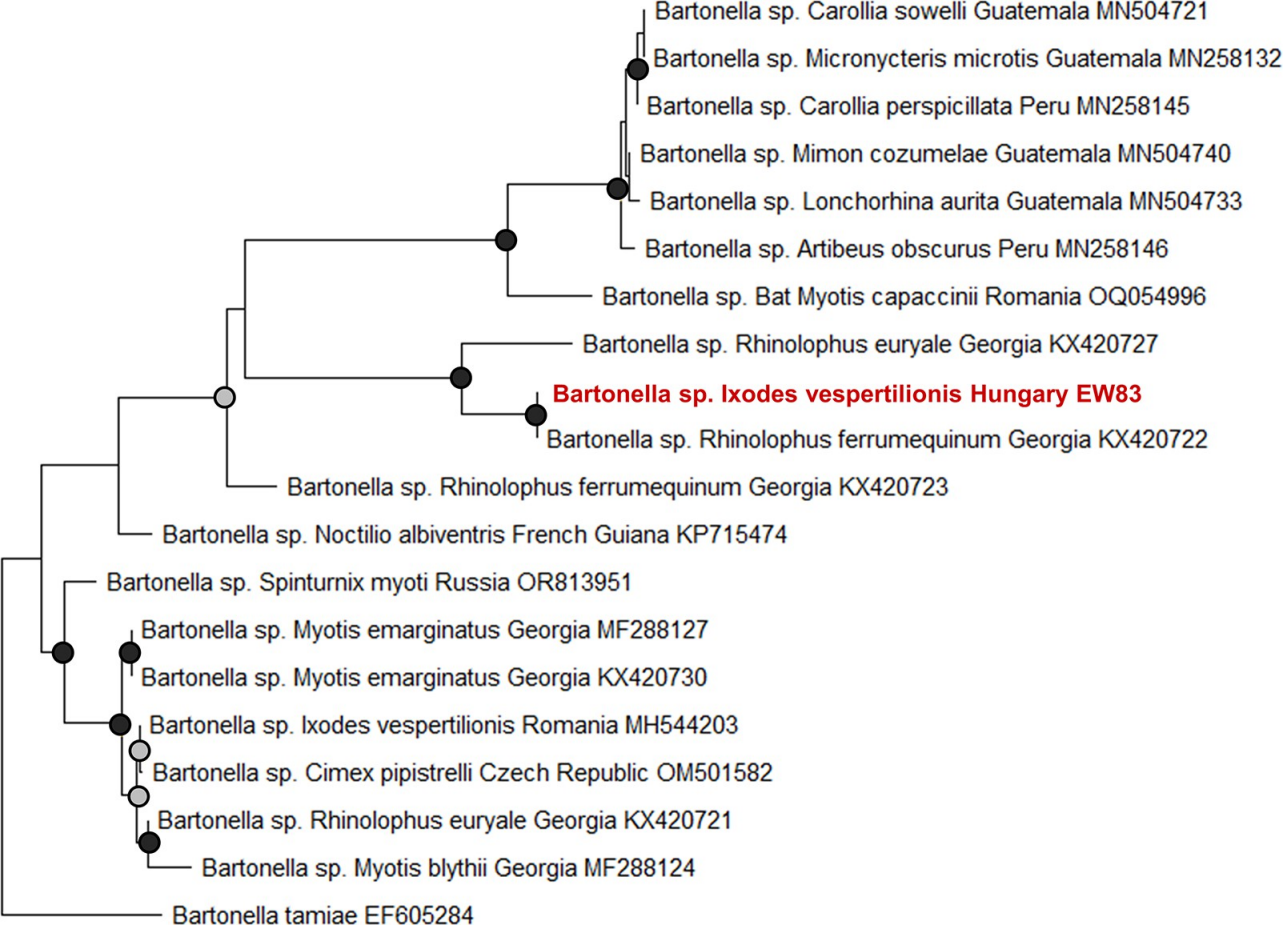
Maximum likelihood tree of *Bartonella* based on internal transcribed spacer (ITS) sequences. The sample acquired during this study is indicated in red. GenBank accession numbers are listed in S2 Table. The phylogenetic tree was made with the Hasegawa-Kishino-Yano model with gamma distribution and invariant sites (HKY+G+I) [39], based on 1000 bootstrap replicates. Gray dots indicate bootstrap values 0.7–0.9, whereas black dots indicate values >0.9, values <0.7 are not indicated.

Bacterial pathogen screening detected the presence of *Bartonella* sp. in a single *I*. *vespertilionis* (n = 1, EW83, Hungary) in the nymph stage that had fed on *R*. *ferrumequinum* (Figs 2 and 3). Sequence data showed the highest similarity with a *Bartonella* sequence found in *R*. *ferrumequinum* in Georgia (KX420722, percentage identity: 100%, query coverage: 97%) [44] (Fig 4).

**Fig 5:**
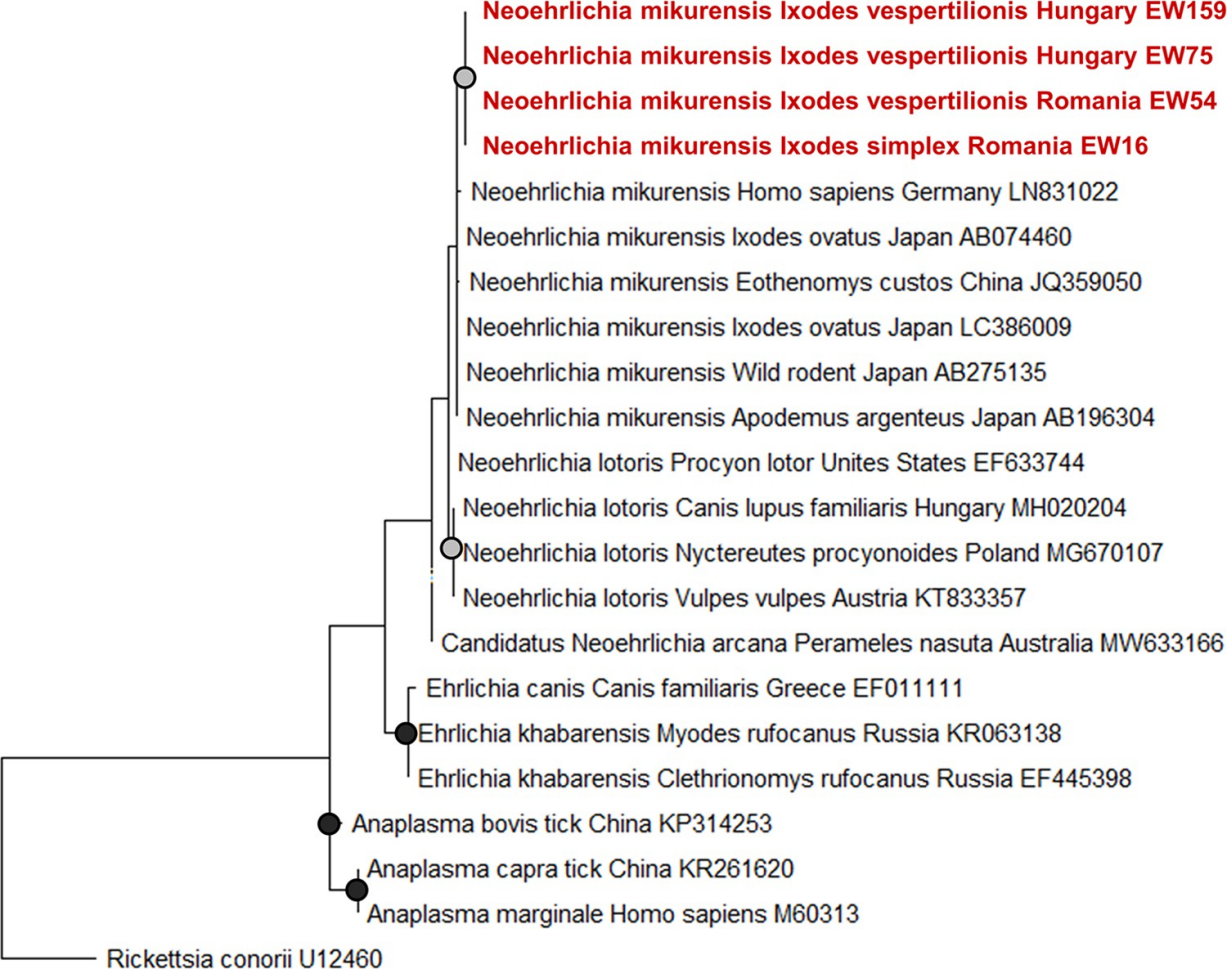
Maximum likelihood tree of Anaplasmataceae sequences based on 16S ribosomal RNA (16S rRNA) gene fragment. Samples acquired during this study are indicated in red. GenBank accession numbers are in S2 Table. The phylogenetic tree was made with the Kimura 2-parameter model [38], based on 1000 bootstrap replicates. Grey dots indicate bootstrap values 0.7–0.9, whereas black dots indicate values >0.9, values <0.7 are not indicated.

We detected *Neoehrlichia mikurensis* in both *I*. *simplex* (n = 1, EW16, Romania) associated with *M*. *schreibersii* and *I*. *vespertilionis* (n = 3, EW54, Romania; EW75, EW159, Hungary), previously fed on *C*. *lupus familiaris*, *M*. *schreibersii*, and *M*. *myotis* (Fig 5). Positive samples for *N*. *mikurensis* showed the highest similarity to *N*. *mikurensis* sequences found in *I*. *ovatus* in Japan (LC386009, percentage identity: 98.7%, query coverage: 100%) [45]. All samples were negative for Anaplasmataceae, *Borrelia burgdorferi* s.l., *Rickettsia* spp., and piroplasms. An additional 15 sequences showed no similarity to known bacteria or matched with uncultured bacterial isolates in GenBank (Accession numbers: PQ444043—PQ444051, PQ444181—PQ444185), therefore their identity could not be determined (Figs 2 and 3).

We detected the presence of *Wolbachia* sp. in *I*. *ariadnae* (n = 9, EW29-30, EW39, EW131-136, Hungary) associated with various bat species, including *M*. *blythii*, *M*. *myotis*, and *M*. *nattereri* (Figs 2 and 3). *Wolbachia* positive samples showed high similarity (98.99–99.66%) to sequences acquired from *Aedes aegypti* (MN046762).

## Discussion

This is the first study providing molecular evidence that bat-associated ixodid ticks opportunistically feed on several non-bat hosts, such as dogs, horses, and wild boars. In addition, *N*. *mikurensis*, was also detected in bat-ticks for the first time, which is a zoonotic bacterial pathogen, often found in rodents serving as reservoirs [46]. The presence of *N*. *mikurensis* was recently described from bats of the species *M*. *schreibersii* [47]. In our samples, two bat tick species (*I*. *simplex*, *I*. *vespertilionis*) and three hosts (*C*. *lupus familiaris*, *M*. *schreibersii* and *M*. *myotis*) were associated with this pathogen. Importantly, dogs are susceptible to the infection and disease caused by *N*. *mikurensis* [48]. These results suggest that bat-associated ticks may play a role in the transmission and maintenance of this pathogen, not only between bat hosts but among other domestic and wildlife species. However, this probably has moderate epidemiological significance, similar to the role of the hedgehog tick (*I*. *hexagonus*), which is taxonomically closely related to bat-associated ticks (subgenus *Pholeoixodes*) and usually carries *N*. *mikurensis* with low prevalence [49]. Until the frequency of non-bat host feeding behavior of bat-associated ticks is quantified and their vectorial capacity is determined, their epidemiological significance cannot be fully assessed.

We found a number of cases, from several geographical locations, where bat-ticks fed on domestic (probably stray) dogs in Romania, whereas no such data were found in Hungary. The presence of free roaming owned or stray dogs is a considerable public and animal health and welfare issue in Romania, due to the lack of management and control of these animals [50]. Free roaming dogs are known to inhabit mines and caves where PCR-positive ticks in this study were collected in Romania. While sharing the same habitat, bat-associated tick and dog contacts are more than likely, however its frequency is unknown. On the contrary, we did not detect domestic dog DNA in bat ticks from Hungary, which is probably attributable to the scarcity of stray dogs at the collection sites in that country; nevertheless, some instances have been documented previously [51]. The presence of a wildlife host, the wild boar in a tick sample from Hungary, suggests that ticks may also feed on other mammals that visit these caves [51]. Additionally, humans, such as cavers and researchers, occasionally visit these caves and may be exposed to these ticks, therefore caution is advised [28].

Furthermore, one *I*. *vespertilionis* tick was positive for horse DNA. Bats such as *M*. *emarginatus* are known to prefer horse stables that are rich in flying insects as roosting and feeding sites [52] and this bat species is a host of *I*. *vespertilionis* [23]. This finding adds to the already documented epidemiological connections between bats and horses, all of which might be relevant to the transmission of zoonotic pathogens (e.g., Hendra virus) between these hosts [53].

*Bartonella* species have been widely described from bats and associated ectoparasites, representing a wide range of genotypes [8,54]. We identified a single *I*. *vespertilionis* tick infected with these bacteria, with high similarity to a *Bartonella* infection from a *R*. *ferrumequinum* tested in Georgia (KX420722) [44]. The infected *I*. *vespertilionis* tick contained *R*. *ferrumequinum* DNA, and it was collected in Hungary, indicating that this *Bartonella* genotype may show a wide geographical range in this bat host species. *Bartonella* spp. occasionally exhibit a relatively high prevalence in bat-associated ticks, such as 11% prevalence in *I*. *ariadnae* and 4% in *I*. *vespertilionis* [11]. In our work, the presence of pathogen DNA may result from the blood meal source of the previous life cycle stage (e.g. in males), however the vectorial potential of ticks (implying transstadial survival of the relevant pathogen) cannot be excluded. These results also suggest that males could be useful to explore feeding behavior of previous life stages, as vertebrate DNA and pathogens could also be detected in them even though they are not known to feed on hosts as adults. Host DNA detection from previous life-stages has been previously demonstrated in other tick species [55]. Nevertheless, it is still unclear whether males of these species feed during adult life stage, but so far none has been found on bats [23]. Additionally, the maintenance of pathogens across life stages may suggest a greater risk of cross-species transmission and indicate the vectorial potential of these ticks. However, the presence of pathogens in field-collected ticks does not provide evidence of vectorial capacity, as they may simply originate from the host’s blood meal. Additionally, we found *Wolbachia* only in *I*. *ariadnae*, however, the source and the role of these bacteria are currently unknown and could derive from hyperparasites, or may serve as endosymbionts or pathogens in tick hosts [56].

Tick life stages can significantly impact pathogen prevalence, vectorial potential, and their role in disease ecology. In this study, we primarily focused on adult females, though we also tested a larva, nymphs, and adult males. As a result, our conclusions are mainly centered on the role of females. However, since we detected a single *Bartonella* infection in a nymph, but not in adults, future research should explore the role of immature life stages in pathogen transmission between bats and other species.

Overall, our results reveal a potentially important aspect of wildlife–domestic animal–human interactions, in this case with the role of cave-visiting stray dogs and stabled horses, that may serve as bridge hosts in the transmission of bat-associated pathogens. However, two of the tick species studied had small sample sizes, and the methods used here cannot accurately determine the frequency of non-bat feeding. Further surveillance studies and experiments would provide valuable information for quantifying tick feeding behavior. Thus, future research should also address these aspects. In light of the above findings, no taxonomic group of bat ectoparasites is known to be shared by bats and other mammals to the extent as bat ticks. Knowing that bat ticks may also feed on humans, this should be evaluated further as a hitherto underestimated route of zoonotic transmission. Conversely, potential pathogen transmission from domestic animals to bats through tick vectors may represent a bat conservation issue, therefore all vector-borne pathogens detected so far in bat-associated ticks deserve evaluation of their eco-epidemiological significance to see if they may pose a threat to bat populations.

## Supporting information

S1 Table Collection data of bat-associated ticks. (CSV)

S2 Table Primers, PCR conditions and GenBank Accession Numbers of Reference sequences. (DOCX)

S1 Appendix PCR conditions and primers. (DOCX)

S1 Fig Tick sampling locations in Hungary and Romania. Spatial data for the global map was obtained from the Natural Earth dataset using QGIS 3.24 [42,43]. Available at: https://www.naturalearthdata.com. No permission is needed to use Natural Earth https://www.naturalearthdata.com/about/terms-of-use/.
(JPG)
