# Supplementary material for: Bat-associated ticks as a potential link for vector-borne pathogen transmission between bats and other animals
Source: PLoS Negl Trop Dis. 2024 Oct 25;18(10):e0012584. doi: 10.1371/journal.pntd.0012584 (PMC11540221; doi:10.1371/journal.pntd.0012584)
Supplement: S2 Table — (DOCX) [file pntd.0012584.s002.docx]

**S2 Table. Reference sequences obtained from GenBank.**

| Pathogen | Host Genus | Host species | Country | GenBank Accession Number | References |
| --- | --- | --- | --- | --- | --- |
| *Bartonella* sp. | Rhinolophus | ferrumequinum | Georgia | KX420722 | [1] |
| *Bartonella* sp. | Rhinolophus | euryale | Georgia | KX420727 | [1] |
| *Bartonella* sp. | Rhinolophus | ferrumequinum | Georgia | KX420723 | [1] |
| *Bartonella* sp. | Noctilio | albiventris | French Guiana | KP715473 | [2] |
| *Bartonella* sp. | Artibeus | obscurus | Peru | MN258146 | [3] |
| *Bartonella* sp. | Mimon | cozumelae | Guatemala | MN504740 | [3] |
| *Bartonella* sp. | Carollia | sowelli | Guatemala | MN504721 | [3] |
| *Bartonella* sp. | Carollia | perspicillata | Peru | MN258145 | [3] |
| *Bartonella* sp. | Micronycteris | microtis | Guatemala | MN258132 | [3] |
| *Bartonella* sp. | Myotis | capaccinii | Romania | OQ054995 | [4] |
| *Bartonella* sp. | Lonchorhina | aurita | Guatemala | MN504733 | [3] |
| *Bartonella* sp. | Ixodes | vespertilionis | Romania | MH544203 | [5] |
| *Bartonella* sp. | Myotis | emarginatus | Georgia | MF288127 | [6] |
| *Bartonella* sp. | Myotis | emarginatus | Georgia | KX420730 | [1] |
| *Bartonella* sp. | Rhinolophus | euryale | Georgia | KX420721 | [1] |
| *Bartonella tamiae* | Outgroup | Outgroup | Outgroup | EF605284 | [7] |
| *Bartonella* sp. | Spinturnix | myoti | Russia | OR813951 | [4] |
| *Bartonella* sp. | Cimex | pipistrelli | Czech Republic | OM501583 | [8] |
| *Bartonella* sp. | Myotis | blythii | Georgia | MF288124 | [6] |
| *Neoehrlichia lotoris* | Procyon | lotor | Unites States | EF633744 | [9] |
| *Neoehrlichia mikurensis* | Ixodes | ovatus | Japan | LC386009 | [10] |
| *Neoehrlichia mikurensis* | Wild | rodent | Japan | AB275135 | [11] |
| *Neoehrlichia mikurensis* | Apodemus | argenteus | Japan | AB196304 | [12] |
| *Neoehrlichia mikurensis* | Ixodes | ovatus | Japan | AB074460 | [13] |
| *Neoehrlichia lotoris* | Canis | lupus familiaris | Hungary | MH020203 | [14] |
| *Neoehrlichia lotoris* | Nyctereutes | procyonoides | Poland | MG670107 | [15] |
| *Neoehrlichia lotoris* | Vulpes | vulpes | Austria | KT833357 | [16] |
| *Neoehrlichia mikurensis* | Homo | sapiens | Germany | LN831022 | [17] |
| *Neoehrlichia mikurensis* | Eothenomys | custos | China | JQ359050 | [18] |
| *Ehrlichia khabarensis* | Myodes | rufocanus | Russia | KR063138 | [19] |
| *Ehrlichia khabarensis* | Clethrionomys | rufocanus | Russia | EF445398 | [20] |
| *Ehrlichia canis* | Canis | familiaris | Greece | EF011111 | [21] |
| Candidatus *Neoehrlichia arcana* | Perameles | nasuta | Australia | MW633165 | [22] |
| *Anaplasma capra* | tick | Outgroup | China | KR261621 | [23] |
| *Anaplasma bovis* | tick | Outgroup | China | KP314253 | [23] |
| *Anaplasma marginale* | Homo | sapiens | Outgroup | M60313 | [24] |
| *Rickettsia conorii* | Outgroup | Outgroup | Outgroup | U12460 | [25] |

References

1. Bai Y, Urushadze L, Osikowicz L, McKee C, Kuzmin I, Kandaurov A, et al. Molecular survey of bacterial zoonotic agents in bats from the country of Georgia (Caucasus). PLoS One. 2017;12: 1–12. doi:10.1371/journal.pone.0171175

2. Davoust B, Marié J-L, Dahmani M, Berenger J-M, Bompar J-M, Blanchet D, et al. Evidence of Bartonella spp. in Blood and Ticks (Ornithodoros hasei) of Bats, in French Guiana. Vector-Borne Zoonotic Dis. 2016;16: 516–519. doi:10.1089/vbz.2015.1918

3. McKee CD, Bai Y, Webb CT, Kosoy MY. Bats are key hosts in the radiation of mammal-associated Bartonella bacteria. Infect Genet Evol. 2021;89: 104719. doi:10.1016/j.meegid.2021.104719

4. Corduneanu A, Zając Z, Kulisz J, Wozniak A, Foucault-simonin A, Moutailler S, et al. Detection of bacterial and protozoan pathogens in individual bats and their ectoparasites using high-throughput microfluidic real-time PCR. Microbiol Spectr. 2023;11: 1–17.

5. Hornok S, Szoke K, Meli ML, Sándor AD, Görföl T, Estók P, et al. Molecular detection of vector-borne bacteria in bat ticks (Acari: Ixodidae, Argasidae) from eight countries of the Old and New Worlds. Parasites and Vectors. 2019;12: 1–7. doi:10.1186/s13071-019-3303-4

6. McKee CD, Kosoy MY, Bai Y, Osikowicz LM, Franka R, Gilbert AT, et al. Diversity and phylogenetic relationships among Bartonella strains from Thai bats. PLoS One. 2017;12: 1–19. doi:10.1371/journal.pone.0181696

7. Kosoy M, Morway C, Sheff KW, Bai Y, Colborn J, Chalcraft L, et al. Bartonella tamiae sp. nov., a newly recognized pathogen isolated from three human patients from Thailand. J Clin Microbiol. 2008;46: 772–775. doi:10.1128/JCM.02120-07

8. Kejíková R, McKee C, Straková P, Šikutová S, Mendel J, Rudolf I. First detection of Bartonella spp. in bat bugs Cimex pipistrelli (Hemiptera: Cimicidae), Central Europe. Parasitol Res. 2022;121: 3341–3345. doi:10.1007/s00436-022-07668-4

9. Yabsley MJ, Murphy SM, Luttrell MP, Wilcox BR, Ruckdeschel C. Raccoons (Procyon lotor), but not rodents, are natural and experimental hosts for an ehrlichial organism related to “Candidatus Neoehrlichia mikurensis.” Vet Microbiol. 2008;131: 301–308. doi:10.1016/j.vetmic.2008.04.004

10. Taira M, Ando S, Kawabata H, Fujita H, Kadosaka T, Sato H, et al. Isolation and molecular detection of Ehrlichia species from ticks in western, central, and eastern Japan. Ticks Tick Borne Dis. 2019;10: 344–351. doi:10.1016/j.ttbdis.2018.11.010

11. Tabara K, Arai S, Kawabuchi T, Itagaki A, Ishihara C, Satoh H, et al. Molecular survey of Babesia microti, Ehrlichia species and Candidatus neoehrlichia mikurensis in wild rodents from Shimane Prefecture, Japan. Microbiol Immunol. 2007;51: 359–367. doi:10.1111/j.1348-0421.2007.tb03923.x

12. Naitou H, Kawaguchi D, Nishimura Y, Inayoshi M, Kawamori F, Masuzawa T, et al. Molecular identification of Ehrlichia species and “Candidatus Neoehrlichia mikurensis” from ticks and wild rodents in Shizuoka and Nagano Prefectures, Japan. Microbiol Immunol. 2006;50: 45–51. doi:10.1111/j.1348-0421.2006.tb03769.x

13. Kawahara M, Rikihisa Y, Isogai E, Takahashi M, Misumi H, Suto C, et al. Ultrastructure and phylogenetic analysis of “Candidatus Neoehrlichia mikurensis” in the family Anaplasmataceae, isolated from wild rats and found in Ixodes ovatus ticks. Int J Syst Evol Microbiol. 2004;54: 1837–1843. doi:10.1099/ijs.0.63260-0

14. Hornok S, Horváth G, Takács N, Farkas R, Szőke K, Kontschán J. Molecular evidence of a badger-associated Ehrlichia sp., a Candidatus Neoehrlichia lotoris-like genotype and Anaplasma marginale in dogs. Ticks Tick Borne Dis. 2018;9: 1302–1309. doi:10.1016/j.ttbdis.2018.05.012

15. Hildebrand J, Buńkowska-Gawlik K, Adamczyk M, Gajda E, Merta D, Popiołek M, et al. The occurrence of Anaplasmataceae in European populations of invasive carnivores. Ticks Tick Borne Dis. 2018;9: 934–937. doi:10.1016/j.ttbdis.2018.03.018

16. Hodžić A, Cézanne R, Duscher GG, Harl J, Glawischnig W, Fuehrer HP. Candidatus Neoehrlichia sp. in an Austrian fox is distinct from Candidatus Neoehrlichia mikurensis, but closer related to Candidatus Neoehrlichia lotoris. Parasites and Vectors. 2015;8: 1–4. doi:10.1186/s13071-015-1163-0

17. Grankvist A, Moore ERB, Stadler LS, Pekova S, Bogdan C, Geißdörfer W, et al. Multilocus sequence analysis of clinical “candidatus neoehrlichia mikurensis” strains from Europe. J Clin Microbiol. 2015;53: 3126–3132. doi:10.1128/JCM.00880-15

18. Li H, Jiang JF, Liu W, Zheng YC, Huo QB, Tang K, et al. Human infection with Candidatus Neoehrlichia mikurensis, China. Emerg Infect Dis. 2012;18: 1636–1639. doi:10.3201/eid1810.120594

19. Rar VA, Pukhovskaya NM, Ryabchikova EI, Vysochina NP, Bakhmetyeva S V., Zdanovskaia NI, et al. Molecular-genetic and ultrastructural characteristics of “Candidatus Ehrlichia khabarensis”, a new member of the Ehrlichia genus. Ticks Tick Borne Dis. 2015;6: 658–667. doi:10.1016/j.ttbdis.2015.05.012

20. Rar VA, Pukhovskaya NM, Vysochina NP, Ivanov LI. Detection of a novel Ehrlichia sp. closely related to Ehrlichia ewengii and Candidatus Ehrlichia shimamensis in wild rodents in Khabarovsk region, far east, Russia. Unpubl Obtained from GenBank. 2015.

21. Siarkou VI, Mylonakis ME, Bourtzi-Hatzopoulou E, Koutinas AF. Sequence and phylogenetic analysis of the 16S rRNA gene of Ehrlichia canis strains in dogs with clinical monocytic ehrlichiosis. Vet Microbiol. 2007;125: 304–312. doi:10.1016/j.vetmic.2007.05.021

22. Egan SL, Taylor CL, Banks PB, Northover AS, Ahlstrom LA, Ryan UM, et al. The bacterial biome of ticks and their wildlife hosts at the urban– wildland interface. Microb Genomics. 2021;7. doi:10.1099/mgen.0.000730

23. Sun XF, Zhao L, Wen HL, Luo LM, Yu XJ. Anaplasma species in China. Lancet Infect Dis. 2015;15: 1263–1264. doi:10.1016/S1473-3099(15)00377-1

24. Weisburg WG, Barns SM, Pelletier DA, Lane DJ. 16S ribosomal DNA amplification for phylogenetic study. J Bacteriol. 1991;173: 697–703. doi:10.1128/jb.173.2.697-703.1991

25. Stothard DR, Fuerst PA. Evolutionary Analysis of the Spotted Fever and Thyphus Groups of Rickettsia Using 16S rRNA Gene Sequences. Syst Appl Microbiol. 1995;18: 52–61. doi:10.1016/S0723-2020(11)80448-0
