# Supplementary material for: Bat-associated ticks as a potential link for vector-borne pathogen transmission between bats and other animals
Source: PLoS Negl Trop Dis. 2024 Oct 25;18(10):e0012584. doi: 10.1371/journal.pntd.0012584 (PMC11540221; doi:10.1371/journal.pntd.0012584)
Supplement: S1 Appendix — (DOCX) [file pntd.0012584.s003.docx]

**S1 Appendix. PCR conditions and primers**

For each vertebrate specific COI, Anaplasmataceae 16S rRNA and Piroplasma sp. 18S rDNA PCR reaction, 5 µl of extracted DNA was added to 20 µl of reaction mixture containing 1.0 U HotStar Taq Plus DNA Polymerase (5 U/µl) (Qiagen, Hilden, Germany), 0.5 µl dNTP Mix (10 mM), 0.5 µl of each primer (50 µM), 2.5 µl of 10× Coral Load PCR buffer (15 mM MgCl2 included) and 15.8 µl distilled water (DW). For the Bartonella sp. 16S-23S ITS PCR reaction (~600bp fragment) further 1 µl extra MgCl2 (25mM) was added and only 14,8 µl distilled water (DW) to reach the 20 µl reaction mixture. In the Rickettsia sp. gltA reaction 2,5 µl of extracted DNA was added to 22,5 µl of reaction mixture containing 1.0 U HotStar Taq Plus DNA Polymerase (5 U/µl) (Qiagen, Hilden, Germany), 0.5 µl dNTP Mix (10 mM), 0.5 µl of each primer (50 µM), 2.5 µl of 10× Coral Load PCR buffer (15 mM MgCl2 included) and 18.3 µl distilled water (DW). For the Borrelia burgdorferi s.l. 5S-23S IGS reaction 5 µl of extracted DNA was added to 20 µl of reaction mixture containing 1.0 U HotStar Taq Plus DNA Polymerase (5 U/µl) (Qiagen, Hilden, Germany), 0.5 µl dNTP Mix (10 mM), 1.0 µl of each primer (10 µM), 2.5 µl of 10× Coral Load PCR buffer (15 mM MgCl2 included) and 14,8 µl distilled water (DW).

**Table A.** Primers and details for conventional PCR methods used in this study.

| **Target group** | **Target gene** | **Primer name** | **Primer sequence (5'-3')** | **Amplicon length (bp)** | **Thermocycling profile** | **Reference** |
| --- | --- | --- | --- | --- | --- | --- |
| **Vertebrate barcode** | cox1 | SFF_145f  SFF_351r | GTH ACH GCY CAY GCH TTY GTA ATA AT  CTC CWG CRT GDG CWA GRT TTC C | ~250 | 95 °C for 5 min; 40× (94 °C for 40 s; 56 °C for 1 min; 72 °C for 1 min); 72 °C for 7 min | [1] |
| ***Anaplasmataceae*** | 16S rRNA | EHR16sD  EHR16sR | GGT ACC YAC AGA AGA AGT CC  TAG CAC TCA TCG TTT ACA GC | ~350 | 95 °C for 10 min; 40× (95 °C for 30 s; 55 °C for 30 s; 72 °C for 45 s); 72 °C for 5 min | [2] |
| ***Bartonella sp.*** | 16S-23S ITS | BA325s  BA1100as | CTT CAG ATG ATG ATC CCA AGC CTT CTG GCG  GAA CCG ACG ACC CCC TGC TTG CAA AGC A | ~600 | 95 °C for 5 min; 40× (94 °C for 30 s; 65 °C for 30 s; 72 °C for 50 s); 72 °C for 5 min | [3,4] |
| ***Borrelia burgdorferi s.l.*** | 5S-23S IGS | B5Sborseq  B23Sborseq | GAG TTC GCG GGA GAG TAG GTT ATT GCC  TCA GGG TAC TTA GAT GGT TCA CTT CC | ~450 | 94 °C for 5 min; 10× ‘touchdown’ (94 °C for 20 s, 70 °C for 30 s (dropping 1°C per cycle), 72 °C for 30 s); 40× (94 °C for 20 s, 60 °C for 30 s, 72 °C for 30 s); 72 °C for 7 min | [5] |
| ***Rickettsia sp.*** | gltA | RpCs.877p  RpCs.1258n | GGG GGC CTG CTC ACG GCG G  ATT GCA AAA AGT ACA GTG AAC A | ~380 | 95 °C for 5 min; 40× (95 °C for 20 s; 48 °C for 30 s; 72 °C for 1 min); 72 °C for 5 min | [6] |
| ***Piroplasma spp.*** | 18S rDNS | BJ1  BN2 | GTC TTG TAA TTG GAA TGA TGG  TAG TTT ATG GTT AGG ACT ACG | ~500 | 95 °C for 10 min; 40× (95 °C for 30 s; 54 °C for 30 s; 72 °C for 40 s); 72 °C for 5 min | [7] |

**References**

1. Walker FM, Williamson CHD, Sanchez DE, Sobek CJ, Chambers CL. Species from feces: Order-wide identification of chiroptera from guano and other non-invasive genetic samples. PLoS One. 2016;11: 1–22. doi:10.1371/journal.pone.0162342

2. Brown GK, Martin AR, Roberts TK, Aitken RJ. Detection of Ehrlichia platys in dogs in Australia. Aust Vet J. 2001;79: 554–558. doi:10.1111/j.1751-0813.2001.tb10747.x

3. Maggi RG, Diniz PP, Cadena MB, Breitschwerdt EB. The Use of Molecular diagnostic Techniques to detect Anaplasma, Bartonella and Ehrlichia Species in Arthropods or Patiens. The International Canine Vector-Borne Disease Symposium. 2006. pp. 9–14.

4. Maia C, Almeida B, Coimbra M, Fernandes MC, Cristóvão JM, Ramos C, et al. Bacterial and protozoal agents of canine vector-borne diseases in the blood of domestic and stray dogs from southern Portugal. Parasites and Vectors. 2014;8: 1–8. doi:10.1186/s13071-015-0759-8

5. Heylen D, Tijsse E, Fonville M, Matthysen E, Sprong H. Transmission dynamics of Borrelia burgdorferi s.l. in a bird tick community. Environ Microbiol. 2013;15: 663–673. doi:10.1111/1462-2920.12059

6. Regnery RL, Spruill CL, Plikaytis BD. Genotypic identification of rickettsiae and estimation of intraspecies sequence divergence for portions of two rickettsial genes. J Bacteriol. 1991;173: 1576–1589. doi:10.1128/jb.173.5.1576-1589.1991

7. Casati S, Sager H, Gern L, Piffaretti JC. Presence of potentially pathogenic Babesia sp. for human in Ixodes ricinus in Switzerland. Ann Agric Environ Med. 2006;13: 65–70.
