# Supplementary figures and images for: Bat-associated ticks as a potential link for vector-borne pathogen transmission between bats and other animals
Source: PLoS Negl Trop Dis. 2024 Oct 25;18(10):e0012584. doi: 10.1371/journal.pntd.0012584 (PMC11540221; doi:10.1371/journal.pntd.0012584)

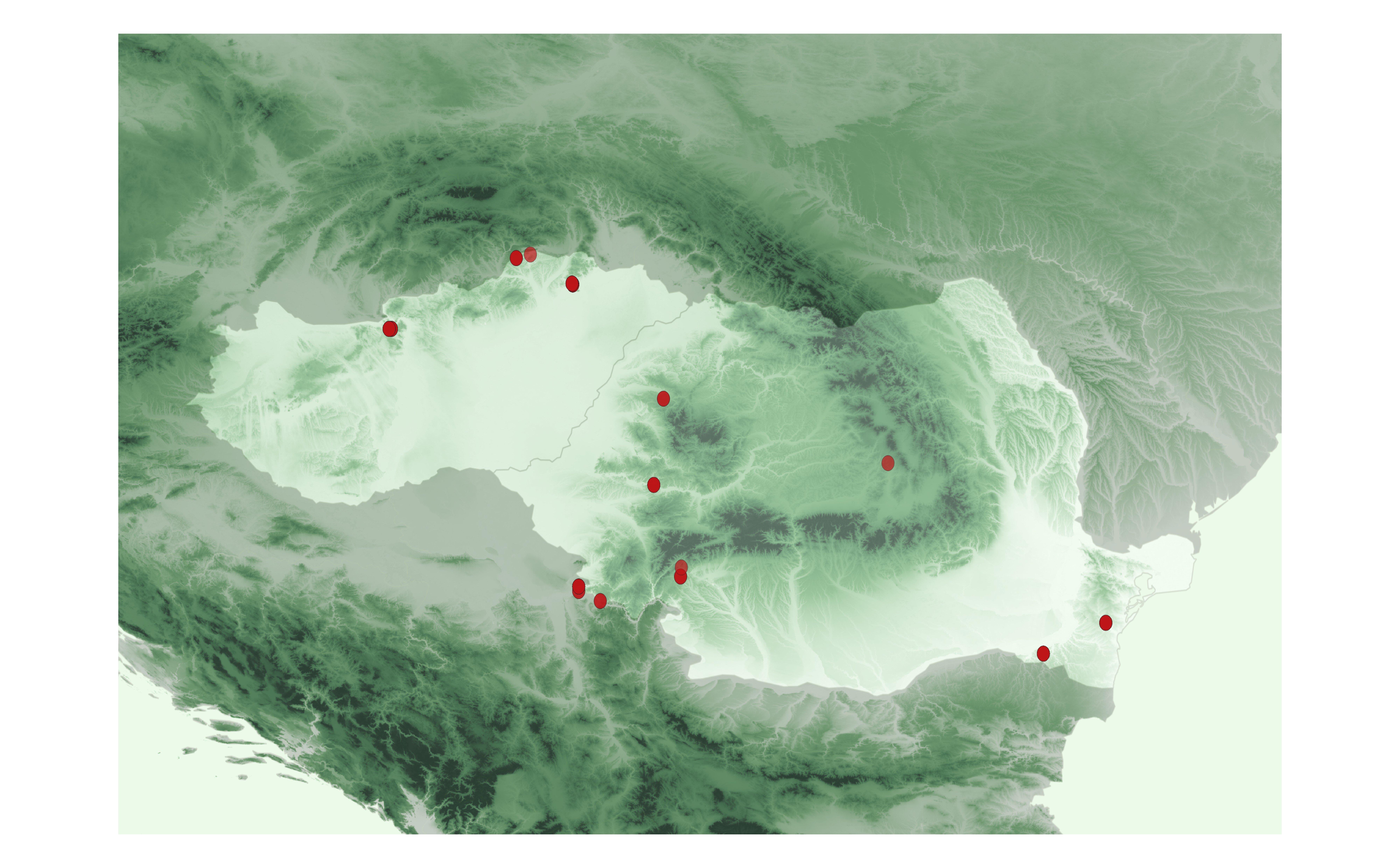

Supplement: S1 Fig — No permission is needed to use Natural Earth https://www.naturalearthdata.com/about/terms-of-use/. (JPG) [file pntd.0012584.s004.jpg]
